# Characterization of the Aeration and Hydrodynamics in Vertical-Wheel^™^ Bioreactors

**DOI:** 10.3390/bioengineering9080386

**Published:** 2022-08-12

**Authors:** Pedro M. Neto, Diogo E. S. Nogueira, Yas Hashimura, Sunghoon Jung, Bruno Pedras, Mário N. Berberan-Santos, Tiago Palmeira, Brian Lee, Joaquim M. S. Cabral, Vitor Geraldes, Carlos A. V. Rodrigues

**Affiliations:** 1 Department of Chemical Engineering, Instituto Superior Técnico, Universidade de Lisboa, Av. Rovisco Pais, 1049-001 Lisbon, Portugal; 2 Associate Laboratory i4HB—Institute for Health and Bioeconomy, Instituto Superior Técnico, Universidade de Lisboa, Av. Rovisco Pais, 1049-001 Lisbon, Portugal; 3 iBB —Institute for Bioengineering and Biosciences, Instituto Superior Técnico, Universidade de Lisboa, Av. Rovisco Pais, 1049-001 Lisbon, Portugal; 4 Department of Bioengineering, Instituto Superior Técnico, Universidade de Lisboa, Av. Rovisco Pais, 1049-001 Lisbon, Portugal; 5 PBS Biotech, Camarillo, CA 93012, USA; 6 Sarspec, Lda, 4400-450 Vila Nova de Gaia, Portugal; 7 CeFEMA—Center of Physics and Engineering of Advanced Materials, Instituto Superior Técnico, Universidade de Lisboa, Av. Rovisco Pais, 1049-001 Lisbon, Portugal

**Keywords:** OpenFOAM, LES, WALE, mesh refinement, partial slip, rigid-lid, Kolmogorov, energy dissipation rate, shear stress, homogeneity, oxygenation, mass transfer, vertical-wheel, Sherwood, human induced pluripotent stem cell, stirred suspension bioreactor, optimization

## Abstract

In this work, the oxygen transport and hydrodynamic flow of the PBS Vertical-Wheel MINI^™^ 0.1 bioreactor were characterized using experimental data and computational fluid dynamics simulations. Data acquired from spectroscopy-based oxygenation measurements was compared with data obtained from 3D simulations with a rigid-lid approximation and LES-WALE turbulence modeling, using the open-source software OpenFOAM-8. The mass transfer coefficients were determined for a range of stirring speeds between 10 and 100 rpm and for working volumes between 60 and 100 mL. Additionally, boundary condition, mesh refinement, and temperature variation studies were performed. Lastly, cell size, energy dissipation rate, and shear stress fields were calculated to determine optimal hydrodynamic conditions for culture. The experimental results demonstrate that the $k_{L}$ can be predicted using $Sh=1.68Re^{0.551}Sc^{\frac{1}{3}}G^{1.18}$, with a mean absolute error of 2.08%. Using the simulations and a correction factor of 0.473, the expression can be correlated to provide equally valid results. To directly obtain them from simulations, a partial slip boundary condition can be tuned, ensuring better near-surface velocity profiles or, alternatively, by deeply refining the mesh. Temperature variation studies support the use of this correlation for temperatures up to 37 °C by using a Schmidt exponent of 1/3. Finally, the flow was characterized as transitional with diverse mixing mechanisms that ensure homogeneity and suspension quality, and the results obtained are in agreement with previous studies that employed RANS models. Overall, this work provides new data regarding oxygen mass transfer and hydrodynamics in the Vertical-Wheel bioreactor, as well as new insights for air-water mass transfer modeling in systems with low interface deformation, and a computational model that can be used for further studies.

## 1. Introduction

Human pluripotent stem cells are generating great hope in the scientific community as they may be used to design innovative therapeutic approaches for numerous health conditions [1]. However, large numbers of cells are required to realize this potential, and therefore, there is the need to establish scalable culture systems for their mass production. Suspension bioreactors allow the monitoring and control of the culture environment, as well as the scale-up of the culture volume without compromising cell quality. The Vertical-Wheel^™^ (PBS Biotech^®^, Camarillo, CA, USA) bioreactors have proven to be successful at ensuring hydrodynamic homogeneity [2] and have been used for the culture of different stem cell types, such as induced pluripotent stem cells [2,3,4] or mesenchymal stem cells [5,6], either on microcarriers or as floating cell aggregates.

In bioreactor cultures, mass transfer of gas and nutrients is often increased by increasing the impeller’s agitation rate, but the associated increase in the shear stress imposed on the free-flowing cell aggregates can impact the cell quality, specifically in terms of differentiation potential, proliferation capacity and phenotype [7,8]. On the other hand, the aggregate size can also be controlled by the shear stress levels. In particular, higher agitation speeds/shear stress levels lead to the formation of smaller aggregates [9], reducing gas and nutrient diffusion limitations to the aggregate core, something that could otherwise cause cell damage and even necrosis by hypoxia [10,11]. Controlling the shear stress should, thus, be performed by manipulating process conditions to achieve an optimal point.

Following this line of thought, computational fluid dynamics (CFD) can be used to study the bioreactor’s hydrodynamic variables, which can be correlated to predict product quality, optimize process conditions and develop scale-up procedures [12,13]. In the case of Vertical-Wheel^™^ bioreactors, studies have been conducted for these purposes using a Reynolds averaged Navier–Stokes (RANS) k−ϵ formulation. However, the aeration process is often neglected and mainly analyzed by empirical studies. This is often because the aeration is easy to measure but hard to simulate, as the interactions between the interface and turbulent structures can strongly influence the mass transfer [14].

To properly study the underlying mechanisms, multiphase models offer a way to simulate the deformation of the gas–liquid interface, with the downside of requiring higher computational costs. However, in cases where surface deformations are low, such as the present one, the effects imposed on the average properties of the bulk and the surface are small enough to be approximated as a rigid lid [14]. Previous works have also studied this possibility using different turbulence models and found that the large eddy simulation (LES) model reproduces the near-surface behavior in wall-bounded rotating flows more accurately [15,16].

In this work, we use the same concepts and simulate the flow and aeration of the PBS Vertical-Wheel MINI^™^ 0.1 for different agitation rates and volumes, comparing the predicted mass transfer coefficients with experimental results. What was found is that the mass transfer coefficient for a given agitation rate, volume, and temperature can be correlated with a modified Sherwood correlation, which uses a dimensionless geometrical term to correlate changes in the working volume. Additionally, we demonstrate how the rigid-lid approximation can offer a simple and inexpensive way to model and predict the oxygenation of the bioreactor and that empirical correlations can be derived from this type of virtual environment to predict product outcomes. Moreover, some effects, such as surface contamination, can significantly impact the mass transfer rate and are difficult to measure or account for [17,18,19]. To correct this issue, we also present a way to calibrate the simulated system allowing us to reproduce the experimentally recorded mass transfer coefficients by dampening the velocity at the interface.

In CFD simulations, the use of a proper mesh is crucial to derive adequate results. Using meshes with different types and zones of refinement can significantly influence the outcome of the simulation, particularly in LES, where turbulent structures are directly resolved up to the size of the mesh cells and, in this case, are essential for a good prediction of the near-surface flow. Because of this fact, we performed a mesh refinement study, where we concluded that the deviation to the theoretical slip condition depends on the refinement level and tends to zero for extremely refined meshes. In addition, another study of virtual nature was performed, in which we tested the impact of the Schmidt on the Sherwood prediction, verifying that the correlation can appropriately predict the mass transfer coefficient for different temperatures.

Finally, the flow inside the bioreactor was characterized, and the velocity oxygen concentration, energy dissipation rate (EDR), Kolmogorov length scale and shear stress fields obtained from the LES model were analyzed to ascertain whether the bioreactor can achieve adequate aggregate suspension and oxygen homogenization, while presenting acceptable shear stress levels, and if so, which range of agitation rates provide the best conditions for cell culture. Taking into account the average variable fields, the results agree with the conclusions presented in other published articles that use a RANS k−ϵ formulation [2,20].

## 2. Materials and Methods

### 2.1. About the PBS MINI^™^

**Figure 1:**
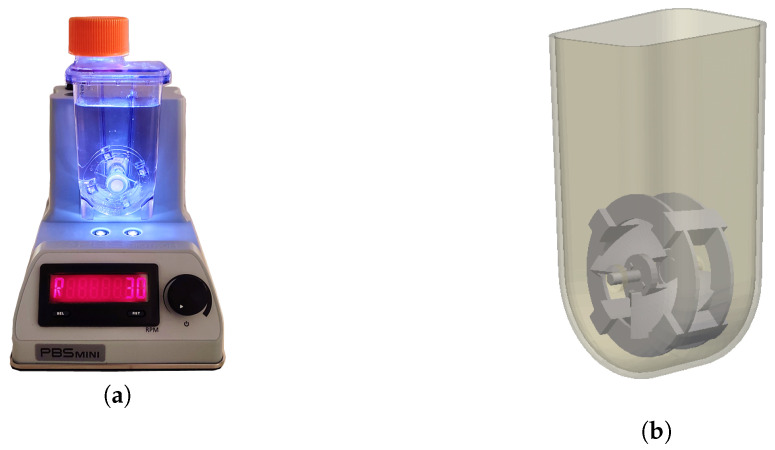
(**a**) Picture of the bioreactor in its base unit. (**b**) Virtual representation of the bioreactor.

The PBS MINI^™^ 0.1 (PBS Biotech^®^, Camarillo, CA, USA) is a single-use bioreactor, constructed from injection-molded polycarbonate, with a working volume between 60 and 100 mL that comprises a vessel with the shape of a lightly funneled box and a U-shaped bottom, containing a vertically oriented impeller wheel, that functions through magnetic agitation induced by the non-disposable base unit in which it rests. The geometry of the wheel presents two curved paddles oriented in the same plane, shaped similarly to elephant-ear impellers, which are supported by two annular structures. Joining these structures, there are four small prisms separated by 90° angles that have magnets embedded in them, allowing the rotation of the whole structure. The agitation rate is controlled by adjusting the dial in the base unit, with the value being displayed next to it. Additionally, the bioreactor has a lid on top that, when opened, puts the medium in contact with fresh air and lets the operator retrieve samples for analysis. This bioreactor is a surface aerated system, and there is no aeration via sparging. A representation of the bioreactor is presented in Figure 1. More information can be consulted in the PBS Biotech official website and in the PBS MINI^™^ user manual [21,22].

### 2.2. Experimental Protocol: Spectroscopy-Based Oxygen Measurements

The experimental protocol used for the oxygen measurements inside the PBS MINI^™^ 0.1 was based on the LED-induced luminescence of a dissolved indicator dye, which when electronically excited, is physically deactivated by the oxygen present in the medium, resulting in a quenching phenomenon and a consequent decrease in the recorded luminescence.

**Figure 2:**
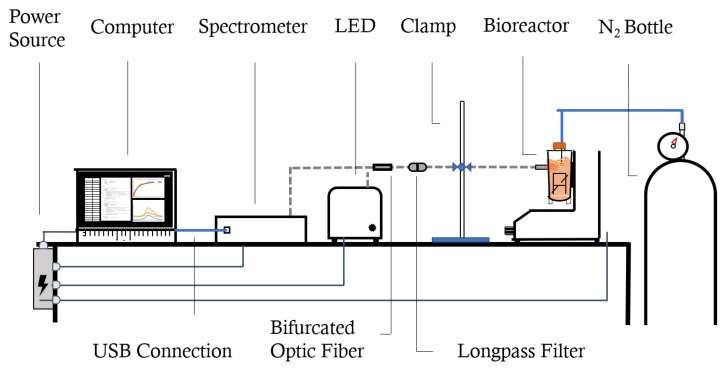
Representation of the experiment set-up (equipment not drawn to scale).

For the set-up, represented in Figure 2, we used a 420 nm LED excitation source and an UV-Vis-NIR FLEX${}^{+}$^™^ spectrometer (Sarspec^®^, Lisbon, Portugal), connected by an 0.4 cm UV-Vis-NIR (ultraviolet, visible and near-infrared) reflectance bifurcated optic fiber with a 530 nm longpass glass filter (SCHOTT^®^, Jena, Germany) inside an in-filter holder connected to another stretch of 0.2 cm optic fiber. The software used in conjunction with the equipment was LightScan^™^ v1.18 (Sarspec^®^, Lisbon, Portugal), for which the run parameters were as follows: 1200 ms of integration time, 10 measure average, and 30 point smoothing.

The indicator solution consisted of 8 mg of tris(2,2${}^{\prime }$-bipyridyl) ruthenium chloride hexahydrate for each 100 mL of deionized water, which was transferred to the bioreactor as needed.

The deaeration was performed at the beginning of the experiments and was carried out by prolonged (minimum of 20 min) nitrogen gas flow through the bioreactor medium, composed entirely of deionized water, until the oxygen reached a steady value close to zero. The aeration of the bioreactor was then performed exclusively with ambient air at a constant agitation rate, up to the point when a steady value was achieved. All of the luminescence measurements were performed without the bioreactor cap and with the optic fiber firmly held against the bioreactor vessel.

### 2.3. Data Treatment

To treat the measured data, we used MATLAB^®^ R2019a v9.6 (The MathWorks Inc., Natick, MA, USA) [23] to adjust the liquid-side mass transfer coefficient, $k_{L}$, via a minimization search.

To determine the oxygen concentration at a given point in time, we integrated the partial differential equation given below:

(1) $$\frac{dC_{O_{2}}}{dt}=k_{L}a\left(C_{O_{2}}^{Sat}-C_{O_{2}}\right)$$

By defining the initial condition as C_O_2__${|}_{t=0}$ = 0, that is, assuming the deaeration was absolute, we obtain the standard concentration profile evolution, where a fixed $k_{L}$ is assumed:

(2) $$C_{O_{2}}\left(t\right)=C_{O_{2}}^{Sat}\left(1-e^{-k_{L}a\times t}\right)$$

For the calculation of the specific area, *a*, the volume was obtained by direct measurement and the contact area was estimated by numerical integration from the virtually generated mesh, which can be consulted in Table 1.

For each time step, the luminescence spectrum was obtained, and the peak intensity (peaking at 608 nm) was determined after the subtraction of the dark spectrum and baseline drift, with the latter being due to equipment-related temperature variations. Then, in order to adapt the inverse relationship between the measured luminescence and the oxygen concentrations (due to the decrease caused by the quenching mechanism), the treated experimental values, $I_{treated\_exp}$, were calculated by subtracting the recorded peaks, $I_{recorded}$, from the luminescence peak at full deaeration, $I_{max}$. Lastly, Equation (4) (analogous to Equation (2)), where $I_{min}$ is the recorded luminescence at saturation, was used to estimate the luminescence values, $I_{calculated}$, from a given mass transfer coefficient. The $k_{L}$ was obtained by minimizing the squared difference between $I_{treated\_exp}$ and $I_{calculated}$:

(3) $$I_{treated\_exp}=I_{max}-I_{recorded}$$

(4) $$I_{calculated}=\left(I_{max}-I_{min}\right)\left(1-e^{-k_{L}a\times t}\right)$$

After obtaining the mass transfer coefficients, a Sherwood correlation was established to predict the system’s behavior relative to the experimental variables:

(5) $$Sh=kRe^{\alpha }Sc^{\beta }$$

Studies indicate that the Sherwood number demonstrates a linear relationship in relation to the Schmidt number to the power of 1/3 [24,25,26,27]. To decrease the amount of correlated variables and avoid correlating experimental errors, we took that value for β as a starting point, and since the temperature variations between experiments were small.

**Figure 3:**
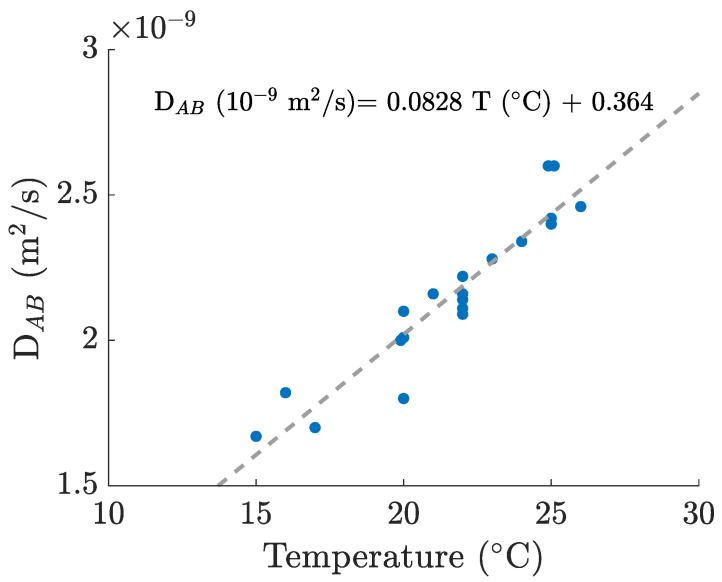
Oxygen–water diffusion coefficients and linear fitting computed from various sources [27,29,30,31,32,33,34,35,36].

For the calculation of the Schmidt number, we computed the kinematic viscosity values [28] for each working temperature, and, for the oxygen diffusion coefficients in water, we used a linear correlation based on several different values from the literature, which can be consulted in Figure 3. This way, we were able to obtain a reasonable estimate for every experimentally recorded temperature (between 21.5 and 25.1 °C).

Finally, changes in volume and surface area were accounted for through a dimensionless number, *G*. This term physically represents the relative length of the wheel diameter to the equivalent liquid height and is given by:

(6) $$G=D\frac{A}{V}$$

To sum up, the following correlation was fitted to the experimental results using the least squares method:

(7) $$Sh=kRe^{\alpha }Sc^{\frac{1}{3}}G^{\gamma }$$

To address its range of validity, the correlation was fitted under Reynolds numbers between 624 and 3172, Schmidt numbers between 366 and 395, and geometrical numbers between 0.651 and 1.08.

### 2.4. Transport Equations and Turbulence Model

The LES model numerically resolves the larger turbulence structures and uses sub-grid scale (*SGS*) models to calculate the turbulence which cannot be directly resolved [37]. To do this, the Navier–Stokes equations are filtered accordingly to the mesh cell size, meaning that the variables are split into super-grid and sub-grid components by spatial filtering:

(8) $$u=\bar{u}+u^{\prime }$$

(9) $$\bar{u}=\int_{D}F\left(x-x^{\prime }\right)u_{i}\left(x^{\prime }\right)dx^{\prime }$$

where *F* is the filter function, generally taking the form of a top-hat filter.

In order to account for the sub-grid stresses, these are split from the diffusion term of the momentum equation. The momentum equation then becomes:

(10) $$\rho \left(\frac{\partial {\bar{u}}_{i}}{\partial t}+\frac{\partial {\bar{u}}_{i}{\bar{u}}_{j}}{\partial x_{j}}\right)=-\frac{\partial \bar{p}}{\partial x_{i}}+\frac{\partial }{\partial x_{i}}\left[\mu \left(\frac{\partial {\bar{u}}_{i}}{\partial x_{j}}+\frac{\partial {\bar{u}}_{j}}{\partial x_{i}}\right)\right]-\frac{\partial \tau_{ij}^{S}}{\partial x_{j}}$$

where ρ, *u*, *p*, and μ are the fluid density, velocity, pressure, and dynamic viscosity, respectively. Then, using the Boussinesq hypothesis, the sub-grid stresses are modeled as:

(11) $$\tau_{ij}^{S}=-2\mu^{t}{\bar{S}}_{ij}\;+\;\frac{\delta_{ij}}{3}\tau_{kk}^{S}$$

in which $\mu^{t}$ is the turbulent viscosity, also referred to as eddy viscosity, and ${\bar{S}}_{ij}$ is the resolved strain rate tensor, given by:

(12) $${\bar{S}}_{ij}=\frac{1}{2}\left(\frac{\partial {\bar{u}}_{i}}{\partial x_{j}}+\frac{\partial {\bar{u}}_{j}}{\partial x_{i}}\right)$$

### 2.5. Sub-Grid Scale Model

To calculate the $\nu^{t}$ values, the wall adapting local eddy-viscosity model (WALE) was employed. It can predict the turbulent viscosity near walls and handle laminar-turbulent transitions better than the traditional Smagorinsky model by using the traceless symmetric part of the square of the velocity gradient tensor to take into account both the rates of strain and rotation:

(13) $$\nu^{t}={\left(C_{w}\Delta \right)}^{2}\frac{{\left(S_{ij}^{d}S_{ij}^{d}\right)}^{\frac{3}{2}}}{{\left({\bar{S}}_{ij}{\bar{S}}_{ij}\right)}^{\frac{5}{2}}+{\left(S_{ij}^{d}S_{ij}^{d}\right)}^{\frac{5}{4}}}$$

In turn, the traceless symmetric part of the square of the velocity gradient tensor, $S_{ij}^{d}$, can be defined by

(14) $$S_{ij}^{d}=\frac{1}{2}\left(\frac{\partial {\bar{u}}_{i}}{\partial x_{k}}\frac{\partial {\bar{u}}_{k}}{\partial x_{j}}+\frac{\partial {\bar{u}}_{j}}{\partial x_{k}}\frac{\partial {\bar{u}}_{k}}{\partial x_{i}}\right)-\frac{\delta_{ij}}{3}{\left(\frac{\partial {\bar{u}}_{k}}{\partial x_{k}}\right)}^{2}$$

### 2.6. Mass Transfer Model

To simulate the mass transfer of the oxygen, the following concentration conservation equation was used:

(15) $$\rho \left(\frac{\partial C_{O_{2}}}{\partial t}+\frac{\partial {\bar{u}}_{i}C_{O_{2}}}{\partial x_{i}}\right)=\left(D_{AB}+D_{AB}^{t}\right)\frac{\partial^{2}C_{O_{2}}}{\partial x_{i}^{2}}$$

where $C_{O_{2}}$ is the oxygen concentration and $D_{AB}$ plus $D_{AB}^{t}$ are the molecular and turbulent diffusion coefficients. The latter is calculated through the turbulent viscosity and the turbulent Schmidt number assumed to be constant and equal to 1.34 given the expected transitional flow and the relatively high Schmidt number [38,39].

(16) $$D_{AB}^{t}=\frac{\nu^{t}}{Sc^{t}}$$

### 2.7. In Silico Protocol

For the simulation of the bioreactor, we assumed an isothermal system and gas–liquid equilibrium based on Henry’s law. The medium was simulated in its entirety as water (as a Newtonian incompressible fluid), and the chosen temperature was 21.0 °C, for which the oxygen–water diffusivity and water kinematic viscosity values are of 2.10 × 10^−9^ m/s and 9.78 × 10^−7^ m^2^/s, respectively. This temperature corresponds to close to the average of the ambient temperatures registered in the first experimental trials.

For this case, where the mass transfer occurs with oxygen in water, the mass transfer resistance in the gas film was considered negligible and, therefore, the $k_{L}$ was approximated as the overall mass transfer coefficient. As for the simulation of the air–water interface, we employed a solid slippery patch with concentration equal to that of the saturation for the arbitrated temperature. Additionally, the initial concentration of the bioreactor was set to zero.

All simulations and meshing were performed in OpenFOAM-8, using 2 virtual machines with 16 Intel (R) Xeon(R) CPU E5-2630 v3 of 2.4 GHz. For the meshing, the bioreactor geometry was provided through a CAD file supplied by PBS Biotech, and the grid was generated by means of the blockMesh and snappyHexMesh utilities. Regarding the rotation of the wheel, it was implemented using a sliding mesh formulation.

In relation to the WALE constants, the ones used were the program’s standards, which represent a good compromise for most flows.

For the boundary conditions (BCs), the no-slip condition was imposed on the solid walls, and for the air–water interface, both the slip and partial slip conditions were tested. The BCs for the turbulent variables were set to “calculated”, as they are computed by the WALE model, and the pressure and concentration BCs were set to zero gradient with the exception of the interface concentration which was set to the oxygen saturation concentration as a fixed value. In the case of the arbitrary mesh interface (AMI) patches, the BC used was always of the type “cyclicAMI”. A summary of the BCs used in the simulations can be consulted in Table 2.

For the time-stepping, the simulation started with a 1.00 × 10^−6^ second value and progressed to the maximum possibly allowed by the Courant–Friedrichs–Lewy (CFL) condition, set to 0.3 as advised for LES simulations [40].

For the time scheme, we employed both the implicit Euler scheme and the Crank–Nicolson scheme with a factor of 0.7, having recorded no significant changes between them.

### 2.8. Post-Processing: Mass Transfer

The calculation of the $k_{L}$ was performed through the ratio between the average mass transfer flux in the air–liquid surface, $\langle N_{O_{2}}\rangle$, and the difference between the saturation concentration, C*_Sat_*, and the average concentration in the bioreactor, $\langle C_{O_{2}}\rangle$:

(17) $$k_{L}=\frac{\langle N_{O_{2}}\rangle }{C_{Sat}-\langle C_{O_{2}}\rangle }$$

As for the local oxygen mass transfer flux in the air-water interface, we used Fick’s first law of diffusion, but to account for the near-surface turbulence, the turbulent diffusivity term was added:

(18) $$N_{O_{2}}=-\left(D_{AB}+D_{AB}^{t}\right){\left.\frac{\partial C_{O_{2}}}{\partial y}\right|}_{surface}$$

These equations were then discretized and implemented in the solver, which can be consulted at https://github.com/Pedro-Miguel-Neto/PBS-MINI-0.1L (accessed on 1 August 2022).

### 2.9. Post-Processing: Turbulent Variables

As described by the turbulent energy cascade, the size of the turbulent structures ranges all the way from biggest to smallest of eddies. If the aggregates can only be influenced by eddies of their size, then it is reasonable to assume that the size of the aggregates is hydrodynamically limited by the size of the smallest eddies. Furthermore, if we accept the assumption that the Kolmogorov eddy length scale theory is valid for transitional flow, we can predict an average size for the aggregates as a function of the EDR, ϵ, and the kinematic viscosity of the medium [15]:

(19) $$\eta ={\left(\frac{\nu^{3}}{\epsilon }\right)}^{\frac{1}{4}}$$

A study has shown that this approximation fits well the experimental data for this type of conditions [41].

To determine ϵ, we used the variable’s definition by inserting it as a programmable function object in the control dictionary:

(20) $$\epsilon =2\nu {\bar{S}}_{ij}{\bar{S}}_{ij}+\epsilon^{SGS}$$

For this, the *SGS* value was directly calculated by the WALE model as such:

(21) $$\epsilon^{SGS}=\frac{C_{\epsilon }\times k^{SGS}\frac{3}{2}}{\Delta }$$

in which *C*_ϵ_ is equal to 1.034 and *k* and Δ correspond to the WALE-calculated kinetic energy and the filter width (the cell’s average length), respectively.

The shear stress felt by the aggregates was estimated by [16]:

(22) $$\tau =\left(\mu +\mu^{t}\right)\sqrt{{\bar{S}}_{ij}{\bar{S}}_{ij}}$$

### 2.10. Post-Processing: Mesh Refinement Quality

To assess the mesh refinement in LES, the following ratio between the *SGS* and overall turbulent kinetic energy was checked:

(23) $$k_{Ratio}=\frac{k_{Resolved}}{k_{Resolved}+k_{SGS}}$$

where the resolved part can be obtained by:

(24) $$k_{Resolved}=\frac{1}{2}\left(\bar{{u_{x}^{\prime }}^{2}}+\bar{{u_{y}^{\prime }}^{2}}+\bar{{u_{z}^{\prime }}^{2}}\right)$$

and in this case, the overline represents a time-average.

When assuming an isotropic nature for the modeled turbulence, it is important that those eddies are short in size, correspondent to the structures that contain the least amount of kinetic energy, i.e., the last 20.0% of the energy cascade. As a rule of thumb, a good mesh should, thus, resolve at least 80.0% of the kinetic energy [42]; by calculating this ratio, we were able to estimate if any parts of the mesh required extra refinement.

### 2.11. Maximum Cell Density

By equaling the oxygen uptake rate (OUR) to the oxygen transfer rate (OTR), a prediction for the maximum cell density, *X*, due to oxygen substrate limitations can be calculated. For this purpose, we can approximate the bioreactor as completely homogeneous and the oxygen concentration in the medium to zero, which is equivalent to assuming that all of the oxygen transferred is being immediately consumed by the cells. Then, by knowing the specific oxygen consumption of the culture at hand, $q_{O_{2}}$, and the operating conditions of the bioreactor, the value for *X* can be given by:

(25) $$X=\frac{k_{L}a\;C_{Sat}}{q_{O_{2}}}$$

## 3. Results

### 3.1. Oxygen Mass Transfer: Experimental Evaluation and Numerical Predictions

As mentioned before, the oxygen mass transfer in the PBS MINI^™^ 0.1 was studied experimentally using the set-up shown in Figure 2. The fittings performed on the experimental results, using Equation (4), showed that an average $k_{L}$ leads to reasonable oxygen concentration predictions over the entire aeration. However, the values tend to be slightly overestimated for the initial time points and underestimated for later time points. This can be observed in the luminescence plots in the Supplementary Data (Figure S1), where initial concentrations are underpredicted and later ones are overpredicted until the values converge to saturation.

**Figure 4:**
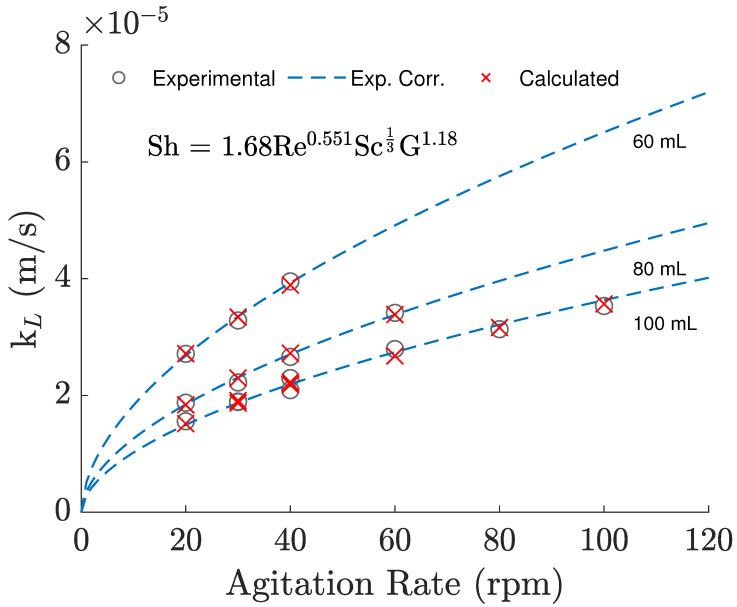
Experimental $k_{L}$ values and experimental correlation predictions against agitation rate. Lines refer to average experimental conditions, while crosses refer to the conditions measured for each respective trial.

Regarding the $k_{L}$ predicted with the correlation from Equation (7), the experimental results were able to be reproduced, as seen in Figure 4, with a maximum error of 4.44%. Analyzing the different sets of data, generated with different working volumes, we can also see that the use of the geometrical term is able to account for changes in the bioreactor volume. Ultimately, the correlated Sherwood numbers displayed linear behavior against the experimental values leading to an R^2^ of 0.992. Moreover, the deviations observed appear to be random, not systematic. Finally, it can be said that changes in temperature could not be empirically verified in an accurate way since temperature differences were practically negligible between experiments, but there is no indication that its variability is being wrongly correlated through a Schmidt exponent of 1/3.

By employing this correlation, we can predict a value for the $k_{L}$, that can then be used to predict a maximum cell concentration through Equation (25), which will depend on the operating conditions and the type of cell culture. For practical purposes, we compared the value obtained by this method with experimental results acquired for the operating conditions of 37 °C, 60 mL, and 30 rpm, where the maximum cell density, observed for human induced pluripotent stem cells after their exponential growth phase, took the value of (2.3 ± 0.2) × 10^6^ cells/mL [43]. Using a range of maximum and minimum values found in the literature for the $q_{O_{2}}$ of the same type of stem cells (1.10 × 10^−16^–1.00 × 10^−18^ mol/cell/s) [44,45,46], we estimated the maximum cell density to be between 2.26 × 10^6^ and 2.49 × 10^8^ cells/mL.

### 3.2. Simulation Results

**Figure 5:**
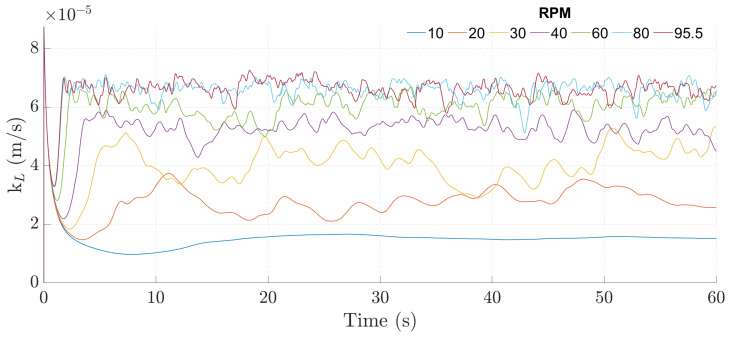
Instantaneous $k_{L}$ over time for the 100 mL and 20 to 95.5 rpm with slip boundary condition cases.

After determining the experimental $k_{L}$ and establishing the correlation, a CFD model was built, as described in Section 2.4, Section 2.5, Section 2.6, Section 2.7, Section 2.8, Section 2.9 and Section 2.10. In the CFD simulations, the behavior of the instantaneous $k_{L}$ demonstrated a steep initial decrease, followed by a rise leading to a quasi-steady state, as shown in Figure 5.

Regarding the values themselves, the simulations overestimated the $k_{L}$ for all agitation rates, with errors reaching upwards to 156% relative to the expected value (calculated through the experimental correlation with the temperature of the simulation).

**Figure 6:**
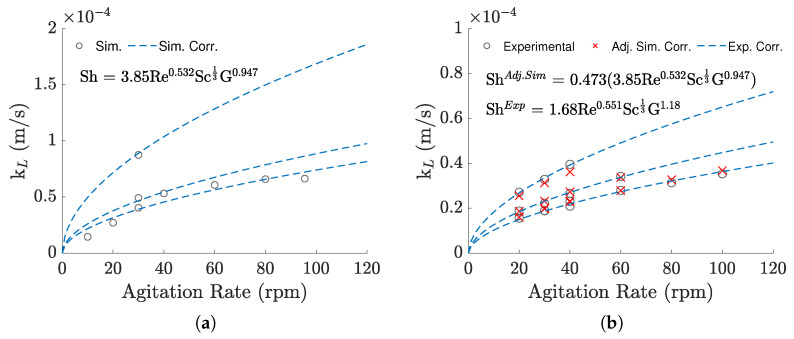
(**a**) Simulation–based $k_{L}$ and simulation-based correlation against agitation rate. (**b**) Comparison between the experimental–based results and the corrected simulation–based correlation, against agitation rate.

Despite the large deviations, the relation of the $k_{L}$ to the agitation rate remained similar (Figure 6a). Because of this, when using the simulation results to fit new constants for Equation (7), the Reynolds exponent only presented relative difference of −3.45% compared to the exponent previously adjusted for the experimental results (which equaled 0.551). However, looking at Figure 6a, we can see that the correlated values under 30 rpm and over 80 rpm are overestimated while the ones between 30 and 80 rpm are underestimated, suggesting that the Sherwood correlation is not able to completely capture the system’s variability the same way it did for the experimental data. It is probably because of this that, although only two simulations for different volumes were taken into account, the geometrical term’s exponent was significantly altered, changing from 1.18 to 0.947.

Regardless, the similarities between the two correlations mean that the error can be greatly reduced by simply changing the linear constant, k, to correct the overpredictions, minimizing the difference between the experimental $k_{L}$ and the one obtained from the correlation. To do this, the experimental conditions (agitation rate, volume, and temperature) for each trial were applied to the correlation obtained by the simulations, seen in Figure 6a, and compared directly to the experimental results in a minimization search, such as before. Figure 6b shows the newly adjusted correlation, with the changes to the linear term represented as a separate constant. As demonstrated in the figure, most values present only slight deviations, with maximum and mean errors of 9.25% and 3.86%, respectively. Table 3 presents a summary of the coefficients derived for the three correlations.

To validate the Schmidt exponent of 1/3, we tested the variation of the Sherwood number by halving the Schmidt, in a separate simulation, for the operational conditions of 100 mL and 30 rpm. The results of that simulation agreed with approximation, demonstrating deviations inferior to 1.00% relative to the predicted value, and confirming that the correlation can be used for different temperatures, thus enabling the prediction of the mass transfer coefficient for temperatures around 37 °C.

Previously, we showed that it was possible to predict a $k_{L}$ in the Vertical-Wheel MINI^™^ 0.1 using an experimental correlation and to derive roughly the same correlation in silico, albeit with a linear deviation. Although the experimental result is more practical at first, the second conclusion can also be important, because it shows that adjusting the same correlation for different cases can be achieved in a virtual environment with relatively low computational costs, requiring less experimental work and possibly lowering research costs. With this in mind, the ability to directly derive the right $k_{L}$ values from the simulations would be of great benefit in order to completely avoid the experimental work.

Having realized that the near-surface velocities were being overestimated, we tried using a different BC for the surface patch, with which the experimental results were able to be directly obtained from the simulations, but still required empirical data to work properly. Concretely, using a partial slip BC, instead of the full slip BC, the experimentally observed value for the 100 mL/20 rpm case was able to be reproduced by tuning the value of the slip coefficient to the value of 12.5%, by trial and error. The slip coefficient acts as a limiter for the surface velocity in relation to that of the adjacent cells and is often used in cases where the flow near the surface deviates from the no-slip condition.

**Figure 7:**
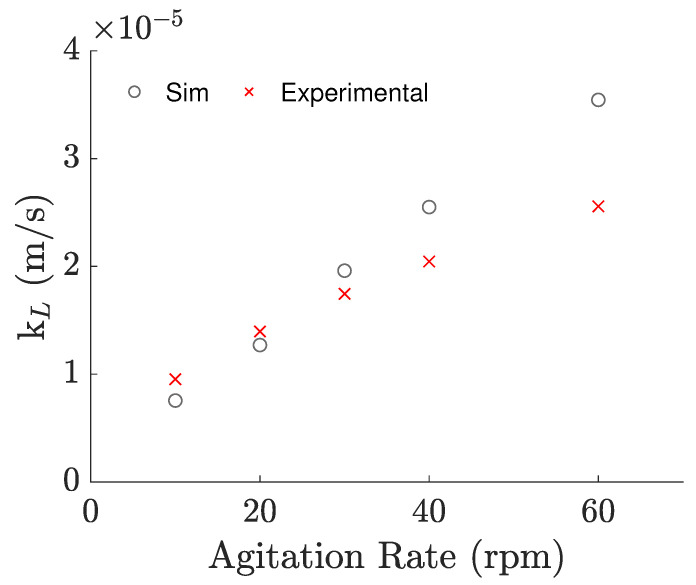
$k_{L}$ values calculated from the experimental–based correlation versus $k_{L}$ values obtained from simulations with a slip coefficient of 12.5%, against agitation rate.

Even though this approach requires an empirical value to tune the slip coefficient, we discovered that the same value can be used to predict the results for neighbor agitation rates with a maximum error of 23.2%, and a mean absolute error of 16.4%, as demonstrated in Figure 7. Unfortunately, the applicability of the same slip coefficient is put into question when considering agitation rates quite different than the one it was adjusted for, or different volumes and/or temperatures, making the empirical-based tuning of this coefficient a practical necessity for the time being. Nonetheless, it is a relative inexpensive way to obtain more accurate predictions for the near-surface flow in cases that apply the rigid-lid approximation and to understand the impact of the near-surface flow in the aeration of the bioreactor and other systems alike.

### 3.3. Mesh Refinement Study

**Figure 8:**
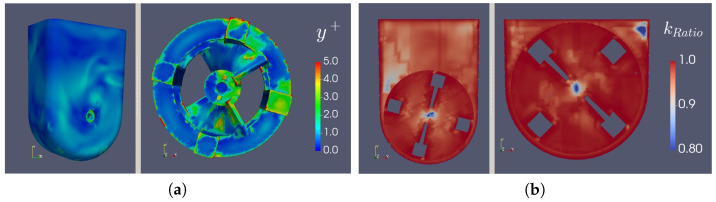
(**a**) y^+^ surface values and (**b**) resolved kinetic energy ratio profiles.

As mentioned before, the quality of the mesh can be of utmost importance in CFD cases. Thus, a mesh refinement study was performed to clarify the influence of this parameter on the simulation results. As Figure 8a demonstrates, we can conclude that for the cases with the most expected turbulence (slip, 100 mL, 95.5 rpm and slip, 60 mL, 30 rpm), more than 80.0% of the turbulent kinetic energy was being resolved by the mesh, and from Figure 8b, we can see that the $y^{+}$ (a dimensionless distance from the wall to the first mesh node, based on local cell fluid velocity) on the wheel, bioreactor walls and the surface was nearly always under 5.00, with values under 1.00 on most of these surfaces. Moreover, in the case of the interface, the $y^{+}$ values all tended to zero. All of these results indicate a fine level of refinement.

**Figure 9:**
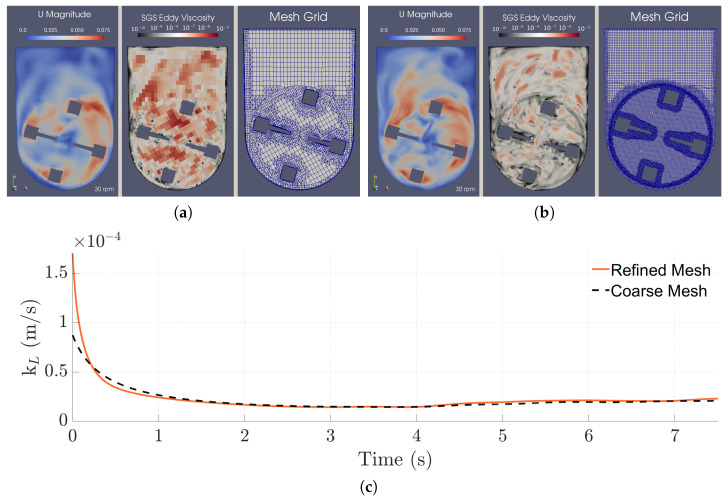
Instantaneous velocity magnitude and *SGS* eddy viscosity fields, and mesh grid in the central xy-plane for the 100 mL/30 rpm case with (**a**) a slip coefficient of 12.5% and with the coarser mesh, and (**b**) a slip coefficient of 5% and with the finer mesh. (**c**) Comparison between the instantaneous $k_{L}$ of both cases.

However, further refining the mesh resulted in a decrease of the calculated $k_{L}$. More specifically, when refined to a much deeper level and throughout all of the control volumes, we found the slip coefficient required to output the same $k_{L}$ to be lower than for the coarser mesh, decreasing from 12.5% to 5.00% and suggesting that the condition tends to the theoretical slip BC for DNS cases. The differences between the coarse and refined cases are presented in Figure 9 (Figure 9a,b show a comparison between the velocity and turbulent viscosity fields, while Figure 9c shows a comparison for the $k_{L}$ values). The *SGS* eddy viscosity profiles show the amount of turbulence that is being modeled by the WALE model. In the case of the coarse mesh, a greater portion of the turbulence is being calculated through the *SGS* model when compared to the refined mesh, where most of the turbulence is being resolved directly through the Navier–Stokes equations.

**Figure 10:**
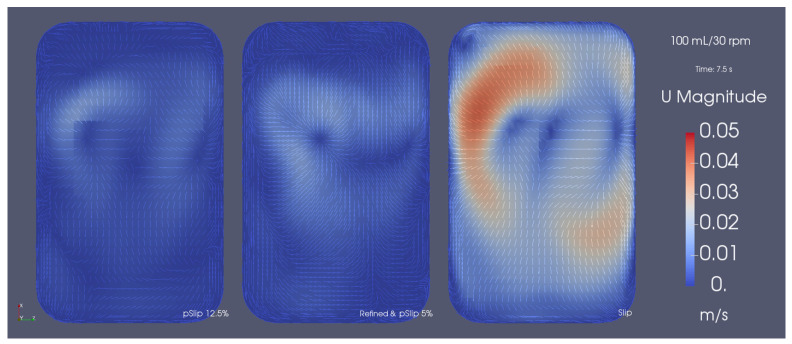
Comparison of the instantaneous surface velocity magnitude field for the slip with coarse mesh (**right**), partial slip 12.5% with coarse mesh (**left**), and partial slip 5.00% with fine mesh cases (**middle**).

Furthermore, when compared to the results of the other simulations, we found the near-surface velocities of the refined mesh to match the ones obtained for the coarse mesh with the partial slip BC, contrasting the overpredicted velocities obtained from the initial simulations, which can be seen in Figure 10.

### 3.4. Mass Transfer Mechanisms

**Figure 11:**
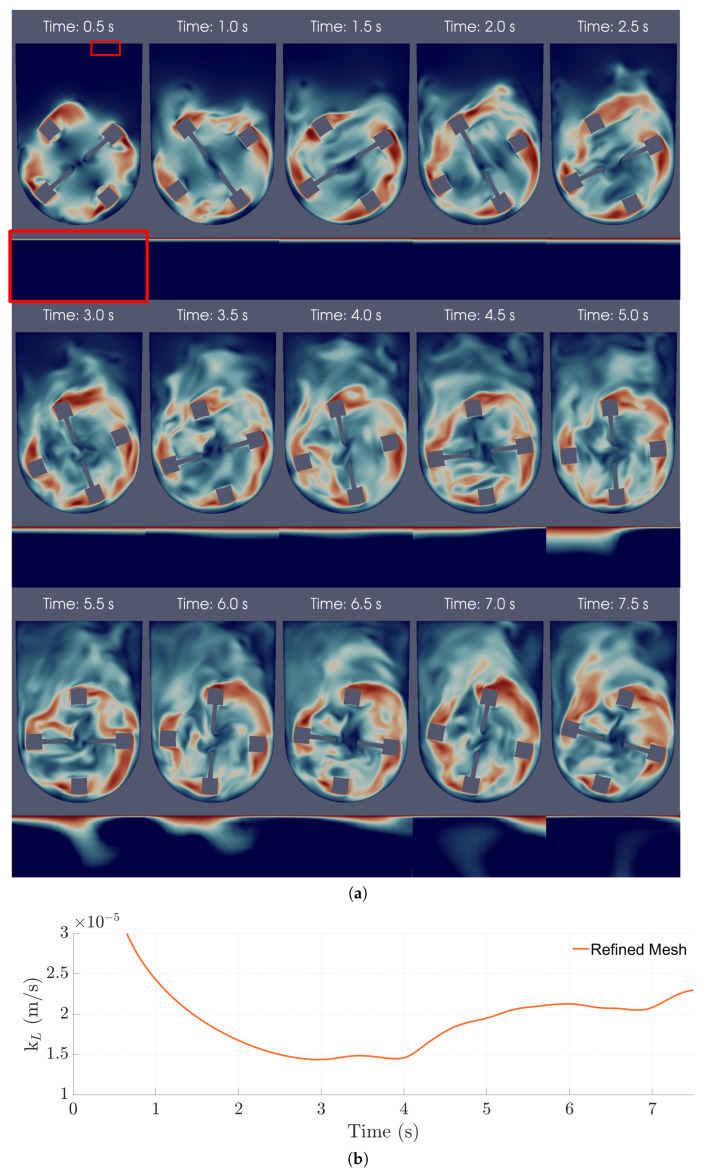
(**a**) Instantaneous velocity magnitude (**top frame**) and near-surface oxygen concentration fields (**bottom frame; amplification**) in the central xy-plane over 7.50 s of operation. The red frame highlights the amplification performed for the oxygen concentration field. (**b**) Instantaneous $k_{L}$ over time of the refined mesh 100 mL/30 rpm case with a slip coefficient of 5.00%.

The aeration mechanism in the bioreactor was studied by examining the oxygen transport during the simulations. In these simulations, surface renewal was confirmed to be the dominant mechanism through visual and numerical observations. In particular, it was possible to observe high-velocity fluid streams coming from the bulk, hitting the interface, and causing the erosion of the concentration boundary layer (CBL), where most of the mass transfer resistance is located (Figure 11a). When this happens, diffusion near the surface is accelerated, leading to higher $k_{L}$ values, as shown in Figure 11b.

### 3.5. Characterization of the Flow

As was previously explored, understanding the flow inside the bioreactor can provide important insights regarding its consequences on the mass transfer through the air–liquid interface. However, mass transfer is constantly occurring throughout the whole working volume, as well as between the medium and the stem cells. Consequently, analyzing how the fluid circulates inside the bioreactor can be of great benefit to bioengineers for understanding, among other things, if the medium is completely homogeneous and whether the suspension of the aggregates is efficient.

**Figure 12:**
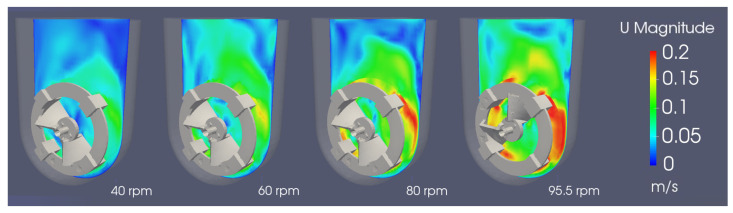
Instantaneous field of velocity magnitude in the central xy-plane for agitations rates between 40 and 95.5 rpm at 60 s.

Figure 12 presents the instantaneous field of the velocity magnitude obtained using three different agitation rates; a comparison with previous studies [2,20], performed with a RANS k−ϵ model, shows consistent results. The magnitude of the velocity profiles is the same, on average, but the flow and turbulent structures are captured in a much more accurate way. This enables a superior understanding of the mixing patterns and the aggregate particle movement inside the bioreactor, as well as the magnitude of the instantaneous hydrodynamic stress to which the cells are exposed, even for the coarser mesh.

**Figure 13:**
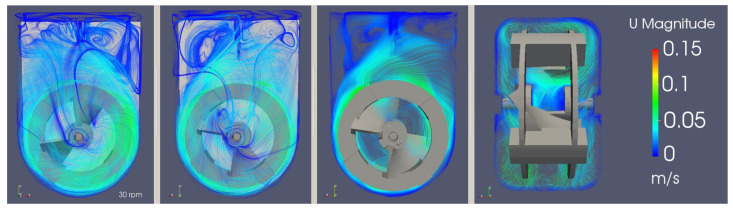
3D and 2D stream tracers of the instantaneous velocity magnitude field for an agitation rate of 30 rpm across different planes.

Looking at Figure 13, the flow can be visually characterized as transitional, with both turbulent and laminar behaviors being spotted. Near the impeller, the flow tends to have higher velocities and eddies of smaller lengths. In contrast, the upper part of the bioreactor presents a free flow zone with lower velocities, but with an increased probability for bigger turbulence structures to form.

**Figure 14:**
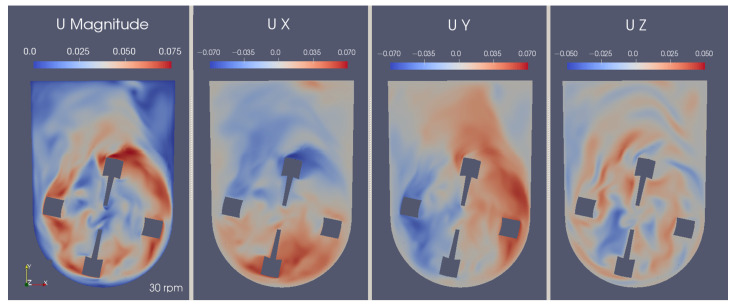
Instantaneous velocity magnitude and directional component fields in the central xy-plane at 7.0 s.

On another note, both axial and radial flow can be spotted. This means that the impeller’s design allows for the passage of fluid in every direction, which, in turn, leads to better homogeneity of gases and suspension of the aggregates. The mechanisms of aggregate suspension become clearer when analyzing the velocity components individually. The U-shaped bottom offers support for sweeping particles to the zones where the upward-directed currents are stronger, effectively reducing the number of stagnant aggregates, as can be observed in Figure 14. Additionally, the longitudinal component (shown in the rightmost profile) demonstrates a lemniscate radial pattern that helps to complete the mixing in a multi-directional way, improving homogenization.

**Figure 15:**
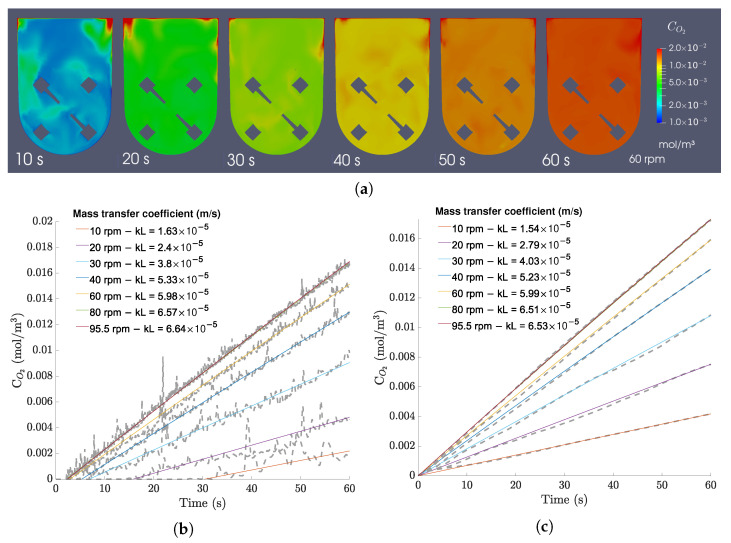
(**a**) Oxygen concentration fields in the central xy-plane for the 100 mL/60 rpm case over 60 s of operation (with a time interval of 10 s). (**b**) Local concentration plots and regressions for all 100 mL cases. (**c**) Average bulk concentration plots and regressions for all 100 mL cases.

In terms of homogeneity of oxygen concentrations in the bioreactor, the oxygen fields are mostly homogeneous soon after starting the agitation, as it is shown in Figure 15a. A comparison between $k_{L}$ values regressed from local cell points and the average bioreactor concentration (Figure 15b,c) shows discrepancies of less than 4.00 × 10^−6^ m/s after an initial delay required for the oxygen to reach those cell points. This means that the average concentration of the bioreactor is roughly equal to the concentration in the majority of the bioreactor, supporting the conclusion that the bioreactor is able to provide efficient mixing and a homogeneous culture environment.

### 3.6. Hydrodynamic Analysis

**Figure 16:**
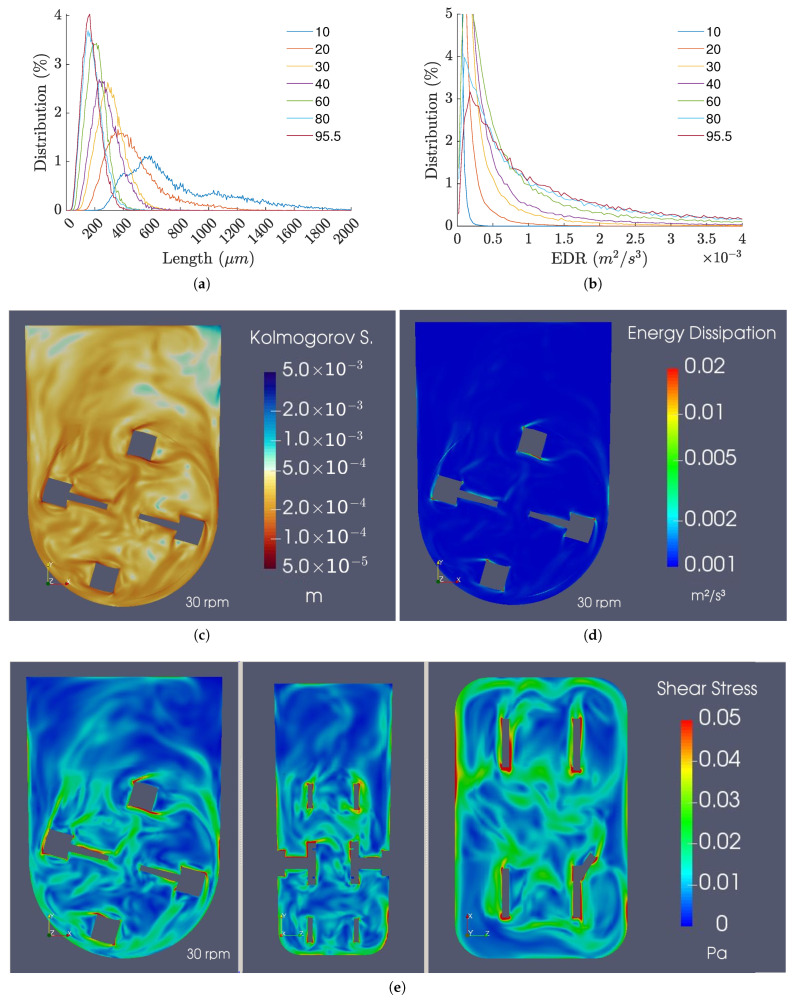
(**a**) Kolmogorov length scale distributions. (**b**) EDR distributions. (**c**) Kolmogorov length scale, (**d**) EDR, and (**e**) shear stress fields for the 100 mL/30 rpm refined mesh case at 7.5 s.

The Kolmogorov length scale is an important parameter usually analyzed to estimate the impact of agitation on animal cell cultures. It was observed that an increase in the agitation rate not only shows a decrease in the Kolmogorov length scale but also an increase in the homogeneity of the size distribution (Figure 16a,c). The size distributions rapidly achieve a steady state for timespans under 10 s, and, for the agitation rates typically used for cell culture, the majority of the predicted sizes varied between 200 and 400 μm.

As for the EDR profiles, Figure 16b,d demonstrate low values across the majority of the control volume, with the exception of the zones near the impeller, where the shear stress is naturally higher. Furthermore, for low to moderate agitation rates, the EDR distributions are uniform and close to the operating range identified for human pluripotent stem cells [20]. However, when agitation rates of 80 rpm and above are used, the EDR values are likely too high to be compatible with stem cell culture due to their exponential increase with agitation rate.

Regarding the instantaneous shear stress profiles, the results demonstrate values relatively larger than the ones anticipated by the averaged results, suggesting that instantaneous stresses can be higher than previously thought. The results of this analysis are presented in Figure 16e.

## 4. Discussion

### 4.1. Experimental Work

In this work, the oxygen mass transfer in Vertical-Wheel^™^ bioreactors was studied in detail for the first time. The oxygen mass transfer coefficient, $k_{L}$, was determined using an optic fiber system, and the results of the luminescence measurements showed that a single $k_{L}$ was insufficient to completely correlate the entire aeration behavior since it was not observed to be constant. Nevertheless, the differences between the observed and predicted oxygen concentration profiles are practically negligible. As a first approach, the $k_{L}$ was obtained through empirical data and used to fit an empirical correlation, modified to account for changes in the volume. The results show that the correlation is satisfactorily adequate to predict the aeration of the bioreactor, capturing the system’s variability, including the changes in volume. When compared to the calculated values, the experimental $k_{L}$ presented only small and random deviations.

The values observed for the aeration of the PBS MINI^™^ revealed a range of $k_{L}$a between 1.58 and 3.71 h^−1^ for 60 rpm and working volumes between 100 and 60 mL. This is reasonably lower compared with other modern bioreactors [47,48,49], making the use of sparging likely necessary for scale-up procedures or high oxygen demanding cultures. The reasons for this lie in the fact that the specific area of the bioreactor is relatively low and the air–water interface is less prone to deformation, contrary to spinner flasks or rocked-bed bioreactors. This was supported by this study, in which decreasing the working volume was the predominant factor for increasing the oxygen mass transfer.

### 4.2. Simulation and Numerical Predictions of the Aeration

In a second part of the study, a CFD model was established for the PBS MINI^™^ 0.1, in order to simulate its operation under different experimental conditions and to characterize the oxygen mass transfer in it. The simulations performed show that the $k_{L}$ (Figure 5) demonstrates a steep decrease for the initial times, followed by an increase and stabilization to a quasi-steady state. This behavior is a response to the formation and disruption of the CBL. The CBL physically represents the thickness of the layer in which most of the mass transfer resistance is located, and as the Schmidt number takes values of 300 and upwards, it is reasonable to assume that the size of the CBL is much smaller than that of the viscous boundary layer; therefore, the aeration process is mostly limited by diffusion between the gas and the liquid phases, and since the air-side mass transfer resistance is negligible, the thickness of the liquid-side CBL is the leading factor when comes to regulating the $k_{L}$ for this system.

At the beginning of the aeration process, the elevated concentration gradient between the surface and the bulk leads to a rapid development of the concentration boundary layer and, as time goes on, the diffusion mechanism becomes less efficient, leading to a slower decrease in the $k_{L}$. This would continue to happen until the $k_{L}$ reached the value of zero, as in the case of a stagnant fluid; however, in the case of this bioreactor, the forced convection generated by the impeller leads to the formation of eddies and wakes that travel to the surface, carrying low concentrations, and causing effective mixing near the surface, momentarily reducing size of the CBL. This phenomenon is generally referred to as surface renewal and is responsible for controlling the rate of mass transfer by enhancing the diffusion process in the interface.

After the stabilization, the values obtained were substantially higher than the ones predicted by the experimental results, which can suggest that the refinement level was not high enough to accurately simulate the mass transfer in the bioreactor. A proof of this was presented in the mesh refinement study, where the $k_{L}$ started to tend to the experimental result as the refinement level increased. On another note, as the refinement level near the surface increased, the initial $k_{L}$ tended to infinity because the surface gradient was now being calculated with a smaller span length.

The simulation results were also used to adjust the constants of the Sherwood correlation. Even though there were differences in the values, the results demonstrated a similar dependency relative to the agitation rate, which allowed us to add a linear term to correct the virtual correlation. This way, predictions can be made under a reasonable margin of error using the CFD-derived correlation. The $k_{L}$ values obtained from the simulations presented deviations from the expected trend, suggesting unaccounted variability that was not observed in the experiments, but an explanation for this could be the fact that there is a distinct behavior for the transition from a mostly laminar regime to a more turbulent one, which is not easily correlated for a wide range of Reynolds and is even harder to account for with the rigid-lid approximation, where the complex interactions between the interface and the forced convection are not accurately portrayed. Further work with more refined meshes and multiphase models could help to answer this question.

Despite this, the $k_{L}$ could be predicted by simulating the surface with a partial slip BC and an adequate slip coefficient. We are convinced that this was possible because the near-surface velocities were being overestimated for the slippery rigid-lid approximation. When using the partial slip condition, the imposed traction forced new eddies to be formed near the surface, imposing a more accurate near-surface velocity profile, which was seen in the refined mesh case, where the turbulence structures can ascend from the bottom of the bioreactor up to the surface. This BC can be useful for predicting the aeration in cases where some variables are difficult to account for, such as impurities in the medium, provided that there is experimental data to tune the coefficient. The usage of this BC with the same slip coefficient should be done with care though, as its applicability is put into question even for moderate variations of the operating conditions.

Addressing the temperature study, temperature differences in the experimental tests were low, making it difficult to properly adjust the Schmidt exponent. Therefore, the standard 1/3 value was taken as an initial guess, but the analysis performed on the Schmidt number demonstrated that this approximation is valid for virtual simulations, which agrees with the experimental data in the literature for most systems that undergo aeration.

### 4.3. Flow and Hydrodynamics

When researched, the characterization of the bioreactor flow displayed transitional properties with both laminar and turbulent characteristics in the flow. Furthermore, it was found that the flow is essentially separated into two zones, the top half of the bioreactor, where flow is free to develop and is influenced by the wakes left by the impeller, and the bottom region, where it is dominated by the forced convection generated by the impeller. Similar to most wall-bounded rotating flows, it is predictable in nature and does not present large eddies; the velocity profiles created by the rotation of the impeller force the direction of the flow and leave little space for the development of turbulence structures other than in the center of the impeller and the trails behind it. It is in the upper part of the bioreactor that most of the larger eddies develop and that most of the interaction between turbulence structures occurs. This is quite beneficial for stem cell environments since these larger eddies ensure efficient mixing in lower velocity zones while maintaining shear stresses low. Another benefit of this bioreactor type is the capacity to maintain cell aggregates in suspension, as the combination between the Vertical-Wheel^™^ and the U-shaped bottom leads to an efficient sweeping of the lower end, converting part of the horizontal kinetic energy into vertical kinetic energy and complementing the ascendant flow generated by the impeller itself. In addition to the radial mixing, by analyzing the longitudinal component of the velocity, it is possible to observe that axial mixing creates a lemniscate pattern, which helps to ensure a faster and better homogenization.

In terms of shear stress, it is important that it remains low, as it can affect cell quality and viability, reducing the process yield. Despite this, shear stress can also limit the aggregate size, which must not be excessively high so as to prevent gas and nutrient diffusion limitations to the core. By approximating the aggregate size to the Kolmogorov length scale, the results suggest that distributions may become reasonably uniform from 30 rpm onward, and as the agitation velocity increases, the size distributions become progressively more uniform. In terms of size, the peaks of the distributions start at 300 μm for 30 rpm and reach 200 and 150 μm for 60 and 95.5 rpm, respectively. In contrast, the increase in the EDR, which is linked to the shear stresses suffered by the aggregates, increases exponentially with agitation rate, displaying considerably high values for agitation rates higher than 60 rpm. Thus, the optimal agitation rate range for 100 mL cultures converges approximately to the same values found in the literature (40 to 80 rpm), as significant distributions for EDR values higher than 0.5 × 10^−3^ m^2^/s^3^ are observed when agitation rates higher than 80 rpm are considered.

Because of the differences in geometry, it is unclear whether or not this bioreactor presents a better hydrodynamic environment for cell culture relative to others, such as spinner flasks. Nonetheless, it was observed that the PBS MINI^™^ is able to operate at lower agitation rates for cell culture, leading to lower shear stress levels while maintaining a high level of cell suspension due to its design [15,16,43].

As a final note, in order to further decrease the EDR inside the bioreactor, we suggest a fine-tuning of the impeller design, for instance, by smoothing the sharper edges of the impeller.

## 5. Conclusions

The results here obtained allow a better understanding of the mixing and mass transfer mechanisms in the Vertical-Wheel^™^ bioreactors, a recently introduced bioreactor configuration, which is being widely adopted in the cell therapy manufacturing field. We can conclude that this bioreactor is, in fact, able to promote efficient mixing and provide homogeneous oxygen concentrations inside the whole vessel. Additionally, a mass transfer correlation is provided to predict the oxygen $k_{L}$ under different operational conditions with this geometry. The work presented not only provides important information for bioprocess design, but also new insights for air-water mass transfer modeling in systems with low interface deformation, and a computational model of the bioreactor that can be used for investigations and optimization, ideally reducing the number of laboratory experiments required.

## Figures and Tables

**Table 1:** Contact area and specific area for different working volumes.

Volume (mL)	Area (cm^2^)	Specific Area (m^−1^)
60.0	15.6	26.1
85.0	16.1	18.9
100	16.4	16.4
105	16.5	15.7

**Table 2:** Simulation boundary conditions.

Field	Air–Water Surface	Solid Walls	Wheel
C_O_2__	fixedValue	zeroGradient	zeroGradient
U	slip/partialSlip	noSlip	movingWallVelocity
P	zeroGradient	zeroGradient	zeroGradient
ν ^t^	calculated	calculated	calculated
k	calculated	calculated	calculated

**Table 3:** Constants for the fitted Sherwood correlations.

Constants	Experimental	Simulation	Adjusted Simulation
k	1.68	3.85	1.82
α	0.551	0.532	0.532
β	0.333	0.333	0.333
γ	1.18	0.947	0.947

## Data Availability

All of the experimental data, OpenFOAM cases, and more can be consulted at https://github.com/Pedro-Miguel-Neto/PBS-MINI-0.1L (accessed on 1 August 2022).

## Supplementary Materials

The following are available online at https://www.mdpi.com/article/10.3390/bioengineering9080386/s1, Figure S1: Luminescence plots derived from the experiments (operating conditions are displayed in the titles).

## Abbreviations

The following abbreviations are used in this manuscript:

AMI	Arbitrary Mesh Interface
BC	Boundary Condition
CBL	Concentration Boundary Layer
CFD	Computational Fluid Dynamics
CFL	Courant–Friedrichs–Lewy
EDR	Energy Dissipation Rate
MDPI	Multidisciplinary Digital Publishing Institute
LES	Large Eddy Simulation
*SGS*	Sub-Grid Scale
LSM	Least Squares Method
OTR	Oxygen Transfer Rate
OUR	Oxygen Uptake Rate
RANS	Reynolds Averaged Navier Stokes
UV-Vis-NIR	Ultraviolet, Visible and Near-Infrared
WALE	Wall Adapting Local Eddy Viscosity

## References

[1] Kim H.J., Park J.S. (2017). Usage of human mesenchymal stem cells in cell-based therapy: Advantages and disadvantages. Dev. Reprod..

[2] Borys B.S., Dang T., So T., Rohani L., Revay T., Walsh T., Thompson M., Argiropoulos B., Rancourt D.E., Jung S. (2021). Overcoming bioprocess bottlenecks in the large-scale expansion of high-quality hiPSC aggregates in vertical-wheel stirred suspension bioreactors. Stem Cell Res. Ther..

[3] Rodrigues C.A., Silva T.P., Nogueira D.E., Fernandes T.G., Hashimura Y., Wesselschmidt R., Diogo M.M., Lee B., Cabral J.M. (2018). Scalable culture of human induced pluripotent cells on microcarriers under xeno-free conditions using single-use vertical-wheel™ bioreactors. J. Chem. Technol. Biotechnol..

[4] Nogueira D.E., Rodrigues C.A., Carvalho M.S., Miranda C.C., Hashimura Y., Jung S., Lee B., Cabral J. (2019). Strategies for the expansion of human induced pluripotent stem cells as aggregates in single-use Vertical-Wheel™ bioreactors. J. Biol. Eng..

[5] de Sousa Pinto D., Bandeiras C., de Almeida Fuzeta M., Rodrigues C.A., Jung S., Hashimura Y., Tseng R.J., Milligan W., Lee B., Ferreira F.C. (2019). Scalable manufacturing of human mesenchymal stromal cells in the vertical-wheel bioreactor system: An experimental and economic approach. Biotechnol. J..

[6] de Almeida Fuzeta M., Bernardes N., Oliveira F.D., Costa A.C., Fernandes-Platzgummer A., Farinha J.P., Rodrigues C.A., Jung S., Tseng R.J., Milligan W. (2020). Scalable production of human mesenchymal stromal cell-derived extracellular vesicles under serum-/xeno-free conditions in a microcarrier-based bioreactor culture system. Front. Cell Dev. Biol..

[7] Gareau T., Lara G.G., Shepherd R.D., Krawetz R., Rancourt D.E., Rinker K.D., Kallos M.S. (2014). Shear stress influences the pluripotency of murine embryonic stem cells in stirred suspension bioreactors. J. Tissue Eng. Regen. Med..

[8] Stolberg S., McCloskey K.E. (2009). Can shear stress direct stem cell fate?. Biotechnol. Prog..

[9] Cherry R., Papoutsakis E. (1986). Hydrodynamic effects on cells in agitated tissue culture reactors. Bioprocess Eng..

[10] Van Winkle A.P., Gates I.D., Kallos M.S. (2012). Mass transfer limitations in embryoid bodies during human embryonic stem cell differentiation. Cells Tissues Organs.

[11] Wu J., Rostami M.R., Cadavid Olaya D.P., Tzanakakis E.S. (2014). Oxygen transport and stem cell aggregation in stirred-suspension bioreactor cultures. PLoS ONE.

[12] Borys B.S., Le A., Roberts E.L., Dang T., Rohani L., Hsu C.Y.M., Wyma A.A., Rancourt D.E., Gates I.D., Kallos M.S. (2019). Using computational fluid dynamics (CFD) modeling to understand murine embryonic stem cell aggregate size and pluripotency distributions in stirred suspension bioreactors. J. Biotechnol..

[13] Muhammad N. (2021). Finite volume method for simulation of flowing fluid via OpenFOAM. Eur. Phys. J. Plus.

[14] Liovic P., Šutalo I.D., Stewart R., Glattauer V., Meagher L. (2012). Fluid flow and stresses on microcarriers in spinner flask bioreactors. Proceedings of the 9th International Conference on CFD in the Minerals and Process Industries.

[15] Berry J., Liovic P., Šutalo I., Stewart R., Glattauer V., Meagher L. (2016). Characterisation of stresses on microcarriers in a stirred bioreactor. Appl. Math. Model..

[16] Ghasemian M., Layton C., Nampe D., zur Nieden N.I., Tsutsui H., Princevac M. (2020). Hydrodynamic characterization within a spinner flask and a rotary wall vessel for stem cell culture. Biochem. Eng. J..

[17] Davies J. (1966). The effects of surface films in damping eddies at a free surface of a turbulent liquid. Proc. R. Soc. Ser. Math. Phys. Sci..

[18] Zappa C.J., Asher W.E., Jessup A.T. (2001). Microscale wave breaking and air-water gas transfer. J. Geophys. Res..

[19] Theofanous T. (1984). Conceptual models of gas exchange. Gas Transfer at Water Surfaces.

[20] Dang T., Borys B.S., Kanwar S., Colter J., Worden H., Blatchford A., Croughan M.S., Hossan T., Rancourt D.E., Lee B. (2021). Computational fluid dynamic characterization of vertical-wheel bioreactors used for effective scale-up of human induced pluripotent stem cell aggregate culture. Can. J. Chem. Eng..

[21] PBS Biotech Offcial Website. https://www.pbsbiotech.com/.

[22] PBS Biotech MINI User Manual. https://www.pbsbiotech.com/uploads/1/7/9/9/17996975/pbs_mini_user_manual_il00266_rev_c.pdf.

[23] MATLAB (2019). 9.6.0.1072779 (R2019a).

[24] Sugano Y., Ratkowsky D. (1968). Effect of transverse vibration upon the rate of mass transfer from horizontal cylinders. Chem. Eng. Sci..

[25] Middleman S. (1998). An introduction to mass and heat transfer: Principles of analysis and design. Eur. J. Eng. Educ..

[26] Bird R.B. (2002). Transport phenomena. Appl. Mech. Rev..

[27] Cussler E.L., Cussler E.L. (2009). Diffusion: Mass Transfer in Fluid Systems.

[28] Kestin J., Sokolov M., Wakeham W.A. (1978). Viscosity of liquid water in the range −8 °C to 150 °C. J. Phys. Chem. Ref. Data.

[29] (2008). Engineering ToolBox. Diffusion Coefficients of Gases in Water. https://www.engineeringtoolbox.com/diffusion-coefficients-d_1404.html.

[30] Oxygen Diffususion Coefficients in Water. http://compost.css.cornell.edu/oxygen/oxygen.diff.water.html.

[31] Holmén K., Liss P. (1984). Models for air-water gas transfer: An experimental investigation. Tellus Chem. Phys. Meteorol..

[32] Welty J., Rorrer G.L., Foster D.G. (2020). Fundamentals of Momentum, Heat, and Mass Transfer.

[33] St-Denis C., Fell C. (1971). Diffusivity of oxygen in water. Can. J. Chem. Eng..

[34] Ramsing N., Gundersen J. (2011). Seawater and gases. Limnol. Ocean..

[35] Chen J., Kim H.D., Kim K.C. (2013). Measurement of dissolved oxygen diffusion coefficient in a microchannel using UV-LED induced fluorescence method. Microfluid. Nanofluid..

[36] Ju L.K., Ho C.S. (1989). Oxygen diffusion coefficient and solubility in n-hexadecane. Biotechnol. Bioeng..

[37] Moukalled F., Mangani L., Darwish M. (2016). The Finite Volume Method in Computational Fluid Dynamics.

[38] Donzis D.A., Aditya K., Sreenivasan K., Yeung P. (2014). The turbulent Schmidt number. J. Fluids Eng..

[39] Gualtieri C., Angeloudis A., Bombardelli F., Jha S., Stoesser T. (2017). On the values for the turbulent Schmidt number in environmental flows. Fluids.

[40] Wolf Dynamics. Tips and Tricks in OpenFOAM. http://www.wolfdynamics.com/wiki/tipsandtricks.pdf.

[41] Le A. (2016). Computational Fluid Dynamics Modeling of Scalable Stirred Suspension Bioreactors for Pluripotent Stem Cell Expansion. Master’s Thesis.

[42] (2016). ANSYS LES Quick Setup Guide, A. Gerasimov, ‘Quick Guide to Setting Up LES-Type Simulations’, Version 1.4. http://www.tfd.chalmers.se/~lada/comp_turb_model/postscript_files/Quick_Guide_to_Setting_Up_LES_version_1.4_for_Lars.pdf.

[43] Nogueira D.E. (2021). Engineering Characterisation of Bioreactors for Human Induced Pluripotent Stem Cell Expansion and Cardiac Differentiation. Ph.D. Thesis.

[44] Varum S., Rodrigues A.S., Moura M.B., Momcilovic O., Easley IV C.A., Ramalho-Santos J., Van Houten B., Schatten G. (2011). Energy metabolism in human pluripotent stem cells and their differentiated counterparts. PLoS ONE.

[45] Zhang J., Nuebel E., Wisidagama D.R., Setoguchi K., Hong J.S., Van Horn C.M., Imam S.S., Vergnes L., Malone C.S., Koehler C.M. (2012). Measuring energy metabolism in cultured cells, including human pluripotent stem cells and differentiated cells. Nat. Protoc..

[46] Turner J., Quek L.E., Titmarsh D., Krömer J.O., Kao L.P., Nielsen L., Wolvetang E., Cooper-White J. (2014). Metabolic profiling and flux analysis of MEL-2 human embryonic stem cells during exponential growth at physiological and atmospheric oxygen concentrations. PLoS ONE.

[47] Gupta A., Rao G. (2003). A study of oxygen transfer in shake flasks using a non-invasive oxygen sensor. Biotechnol. Bioeng..

[48] Zhang H., Williams-Dalson W., Keshavarz-Moore E., Shamlou P.A. (2005). Computational-fluid-dynamics (CFD) analysis of mixing and gas–liquid mass transfer in shake flasks. Biotechnol. Appl. Biochem..

[49] Nikakhtari H., Hill G.A. (2005). Modelling oxygen transfer and aerobic growth in shake flasks and well-mixed bioreactors. Can. J. Chem. Eng..

