# Supplementary material for: Characterization of the Aeration and Hydrodynamics in Vertical-Wheel™ Bioreactors
Source: Bioengineering (Basel). 2022 Aug 12;9(8):386. doi: 10.3390/bioengineering9080386 (PMC9405225; doi:10.3390/bioengineering9080386)
Supplement: Supplementary file 1 [file bioengineering-09-00386-s001.zip › bioengineering-1827303-supplementary-author proof.pdf]

bioengineering

MDPI

Article

Characterization of the Aeration and Hydrodynamics in Vertical-Wheel™ Bioreactors

Pedro M. Neto [1,2,3], Diogo E. S. Nogueira [2,3,4], Yas Hashimura [5], Sunghoon Jung [5], Bruno Pedras [1,2,3], Mário N. Berberan-Santos [1,2,3], Tiago Palmeira [6], Brian Lee [5], Joaquim M. S. Cabral [2,3,4], Vítor Geraldes [1,7,*] and Carlos A. V. Rodrigues [2,3,4,*]

1 Department of Chemical Engineering, Instituto Superior Técnico, Universidade de Lisboa, Av. Rovisco Pais, 1049-001 Lisbon, Portugal

2 Associate Laboratory i4HB—Institute for Health and Bioeconomy, Instituto Superior Técnico, Universidade de Lisboa, Av. Rovisco Pais, 1049-001 Lisbon, Portugal

3 iBB —Institute for Bioengineering and Biosciences, Instituto Superior Técnico, Universidade de Lisboa, Av. Rovisco Pais, 1049-001 Lisbon, Portugal

4 Department of Bioengineering, Instituto Superior Técnico, Universidade de Lisboa, Av. Rovisco Pais, 1049-001 Lisbon, Portugal

5 PBS Biotech, Camarillo, CA 93012, USA

6 Sarspec, Lda, 4400-450 Vila Nova de Gaia, Portugal

7 CeFEMA — Center of Physics and Engineering of Advanced Materials, Instituto Superior Técnico, Universidade de Lisboa, Av. Rovisco Pais, 1049-001 Lisbon, Portugal

* Correspondence: vitor.geraldes@tecnico.ulisboa.pt (V.G.); carlos.rodrigues@tecnico.ulisboa.pt (C.A.V.R.)

Abstract: In this work, the oxygen transport and hydrodynamic flow of the PBS Vertical-Wheel MINI™ 0.1 bioreactor were characterized using experimental data and computational fluid dynamics simulations. Data acquired from spectroscopy-based oxygenation measurements was compared with data obtained from 3D simulations with a rigid-lid approximation and LES-WALE turbulence modeling, using the open-source software OpenFOAM-8. The mass transfer coefficients were determined for a range of stirring speeds between 10 and 100 rpm and for working volumes between 60 and 100 mL. Additionally, boundary condition, mesh refinement, and temperature variation studies were performed. Lastly, cell size, energy dissipation rate, and shear stress fields were calculated to determine optimal hydrodynamic conditions for culture. The experimental results demonstrate that the k_L can be predicted using $Sh = 1.68Re^{0.551}Sc^{\frac{1}{3}}G^{1.18}$, with a mean absolute error of 2.08%. Using the simulations and a correction factor of 0.473, the expression can be correlated to provide equally valid results. To directly obtain them from simulations, a partial slip boundary condition can be tuned, ensuring better near-surface velocity profiles or, alternatively, by deeply refining the mesh. Temperature variation studies support the use of this correlation for temperatures up to 37°C by using a Schmidt exponent of 1/3. Finally, the flow was characterized as transitional with diverse mixing mechanisms that ensure homogeneity and suspension quality, and the results obtained are in agreement with previous studies that employed RANS models. Overall, this work provides new data regarding oxygen mass transfer and hydrodynamics in the Vertical-Wheel bioreactor, as well as new insights for air-water mass transfer modeling in systems with low interface deformation, and a computational model that can be used for further studies.

Keywords: OpenFOAM; LES; WALE; mesh refinement; partial slip; rigid-lid; Kolmogorov; energy dissipation rate; shear stress; homogeneity; oxygenation; mass transfer; vertical-wheel; Sherwood; human induced pluripotent stem cell; stirred suspension bioreactor; optimization

check for updates

Citation: Neto, P.M.; Nogueira, D.E.S.; Hashimura, Y.; Jung, S.; Pedras, B.; Berberan-Santos, M.N.; Palmeira, T.; Lee, B.; Cabral, J.M.S; Geraldes, V.; et al. Characterization of the Aeration and Hydrodynamics in Vertical-Wheel™ Bioreactors. *Bioengineering* **2022**, 9, 386. https://doi.org/10.3390/bioengineering9080386

Received: 5 July 2022
Accepted: 2 August 2022
Published: 12 August 2022

Publisher's Note: MDPI stays neutral with regard to jurisdictional claims in published maps and institutional affiliations.

Copyright: © 2022 by the authors. Licensee MDPI, Basel, Switzerland. This article is an open access article distributed under the terms and conditions of the Creative Commons Attribution (CC BY) license (https://creativecommons.org/licenses/by/4.0/).

Supplementary Data

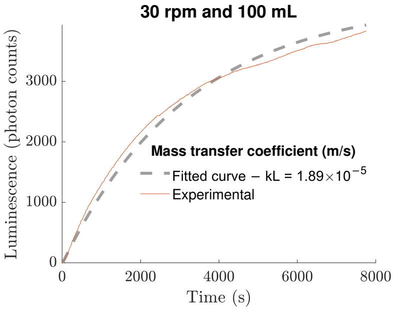

(a)

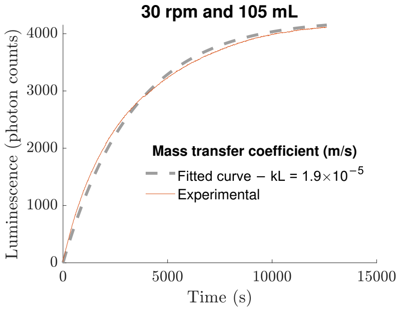

(b)

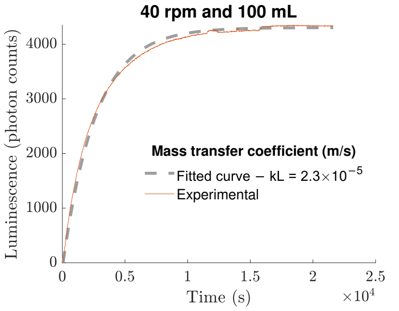

(c)

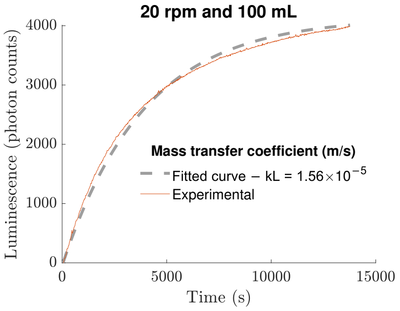

(d)

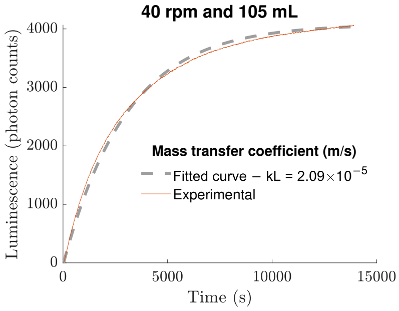

(e)

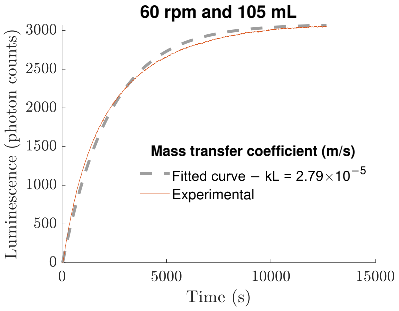

(f)

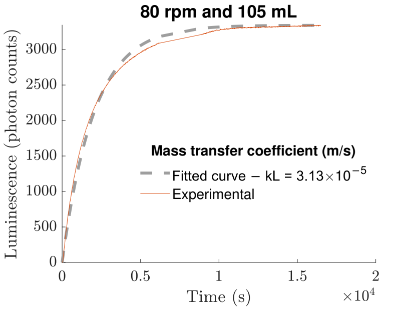

(g)

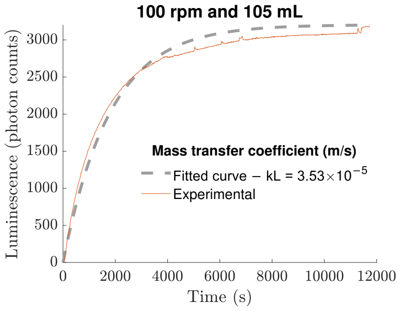

(h)

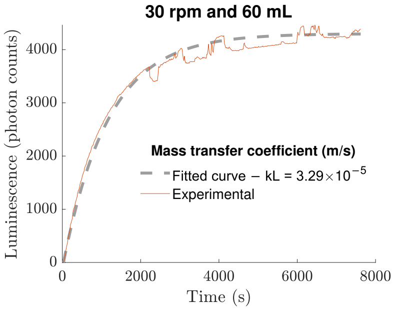

(i)

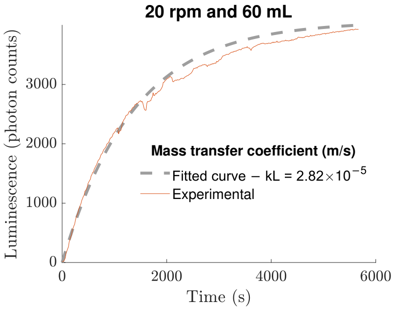

(j)

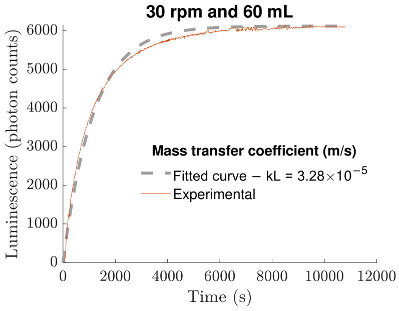

(k)

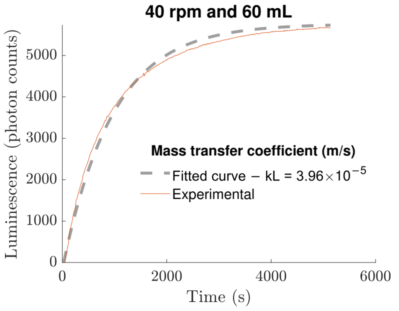

(l)

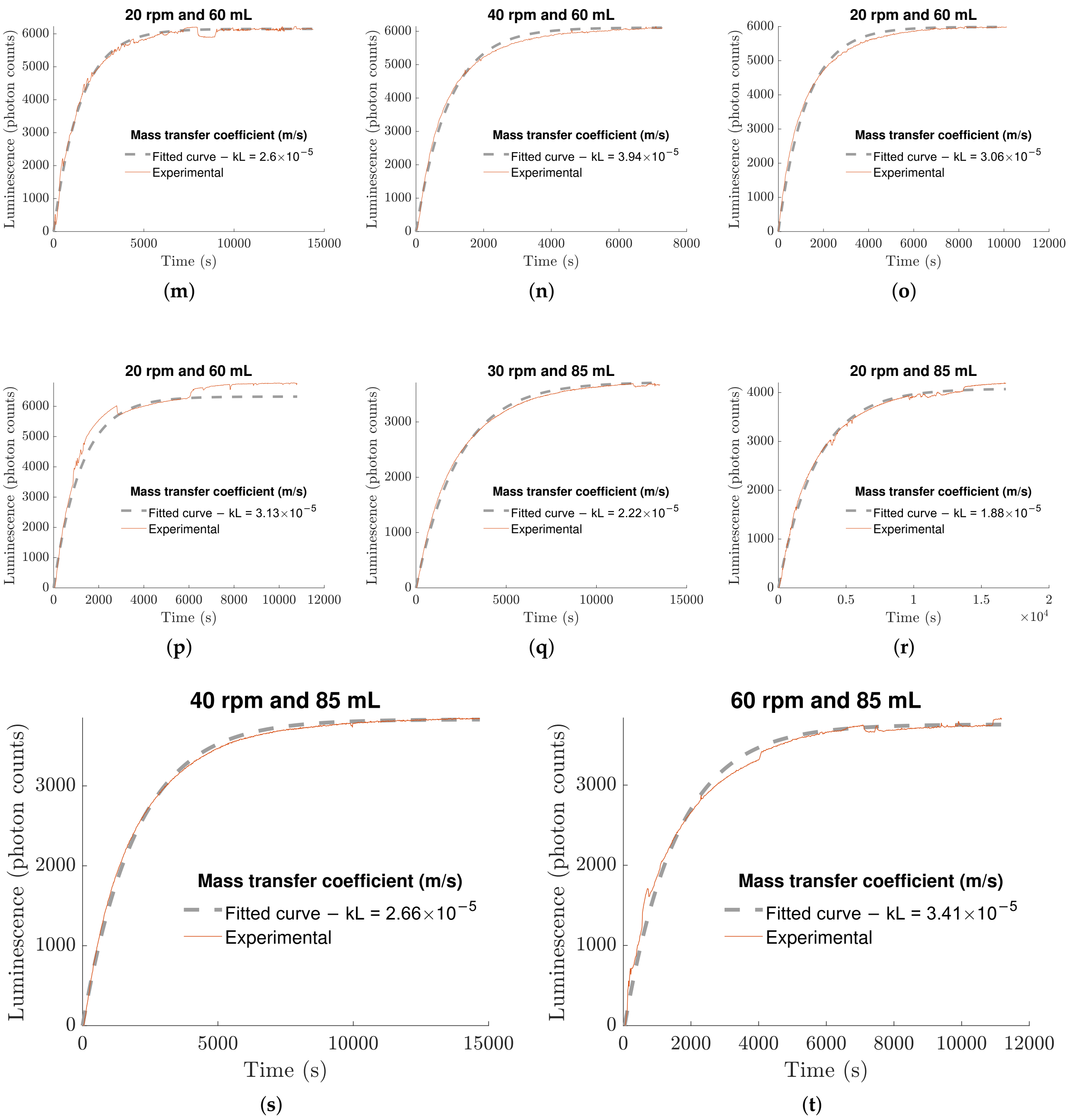

Figure S1. Luminescence plots derived from the experiments (operating conditions are displayed in the titles).
